# Australia’s colourful path to tuberculosis freedom

**DOI:** 10.1186/s13620-023-00244-x

**Published:** 2023-07-25

**Authors:** Ronald J. Glanville

**Affiliations:** 1 Department of Agriculture and Fisheries, Biosecurity Queensland, Brisbane, QLD 4001 Australia; 2 Present Address: Biosecurity Advisory Service, Woodend, VIC 3442 Australia

**Keywords:** Bovine tuberculosis, Eradication, Australia, Case studies

## Abstract

The aim of this paper is to highlight the key lessons learned from Australia’s successful program to eradicate bovine tuberculosis (TB) over a 27-year period from 1970 when the Brucellosis and Tuberculosis Eradication Campaign commenced, through to when TB freedom was declared on 31 December 1997.

As well as discussing the key elements of the national program and its success factors, the author documents a number of case studies and reflects on personal experiences in the far north-west of the state of Queensland during the very difficult latter phases of the program from the mid 1980s and subsequently as State program leader. The late 1980’s was a crucial time in the program leading up to a target declaration of Impending Freedom from TB.

## Background

**Fig. 1:**
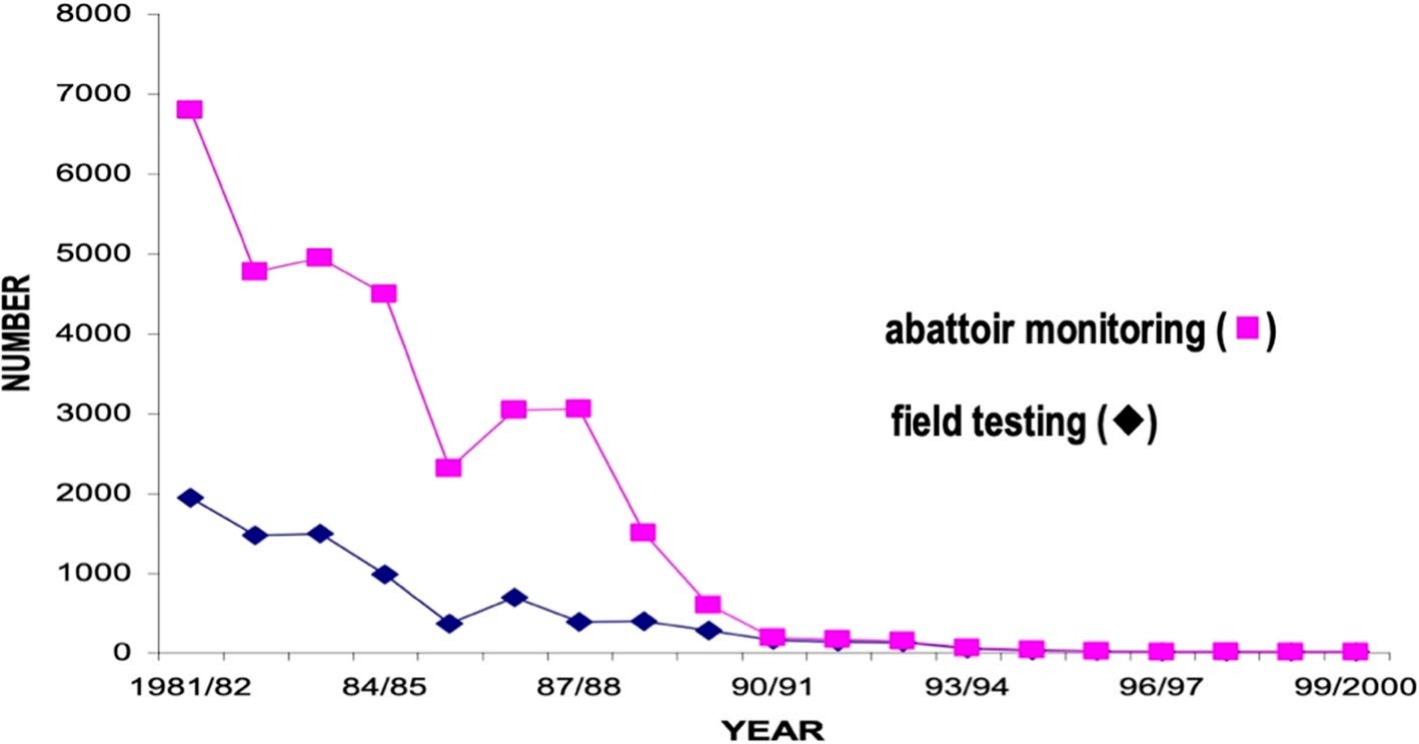
Tuberculosis infection in cattle in Australia (1981–2000), as detected by abattoir monitoring and field testing. Adapted from [2]

Australia eradicated bovine tuberculosis (TB) over a 27-year period from 1970 when the Brucellosis and Tuberculosis Eradication Campaign (BTEC) commenced, through to when TB freedom was declared on 31 December 1997 [1–3]. Figure 1 [2] shows the decline in incidence from both abattoir monitoring and field testing, with the last case detected in 2002 [4]. The original impetus for eradication, apart from cattle productivity benefits and human health concerns, was to ensure ongoing international market access for Australia’s beef production, given that Australia exported around three quarters of what it produced [3]. The major concern was access to the USA market, the largest international customer for beef products at the time. In hindsight, the fear of loss of markets was probably not borne out. However, there is no doubt that the success of BTEC has enhanced the reputation of Australia’s animal health system and had a positive impact on international market access in an indirect way. Further, there are a number of ongoing legacies of BTEC, which are discussed later in this paper.

The key elements of this approximately AUD$1 billion program have been well documented [1, 3, 4]. The most important of these from the author’s perspective are discussed later. However, to provide a feel for how the program operated on the ground, reflections on personal experiences in the far north-west of Queensland during the very difficult latter phases of the program from the mid 1980s are provided.

## TB eradication in North West Queensland

**Fig. 2:**
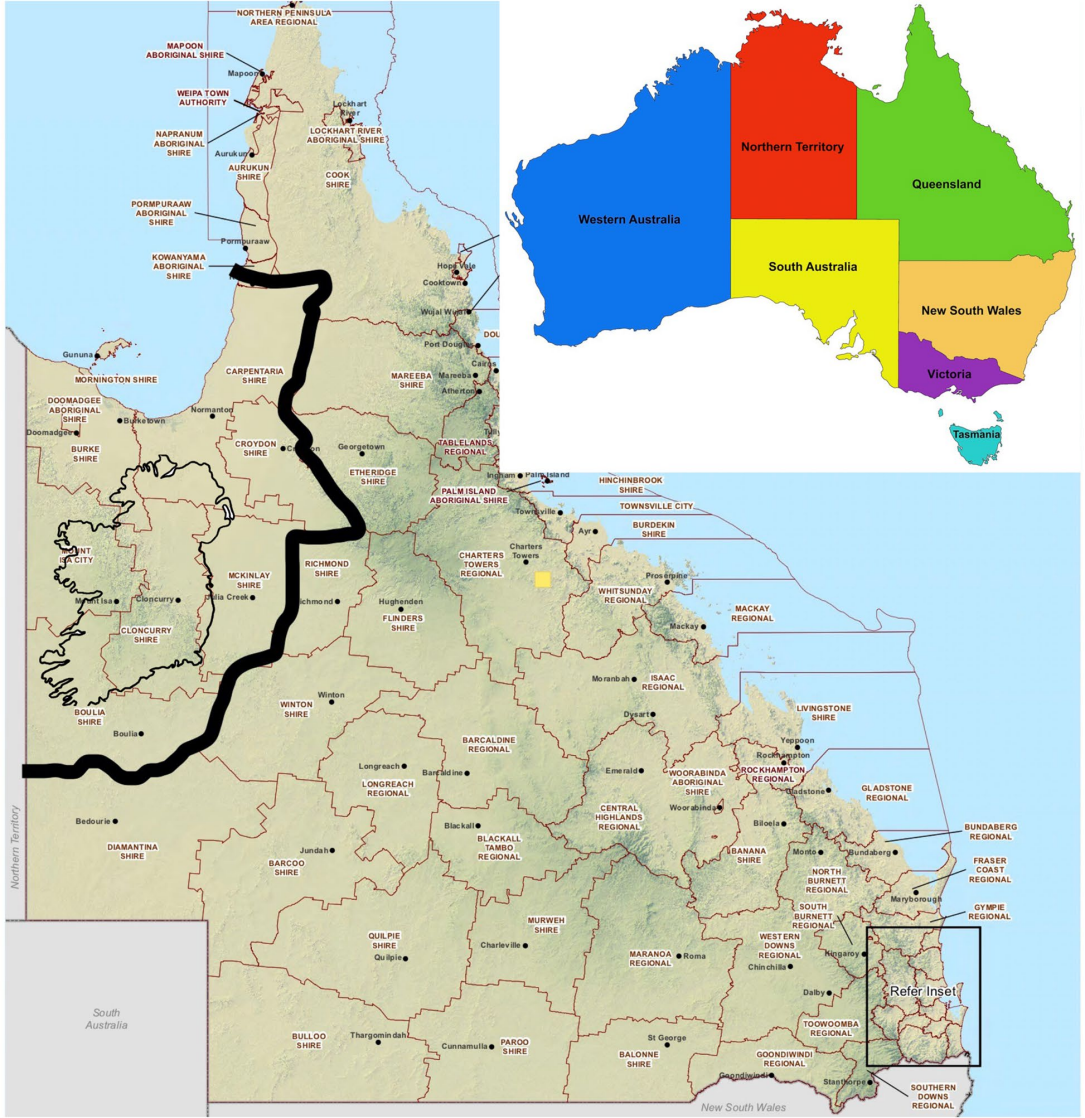
Map of Mount Isa Veterinary Division in 1987, with map of Ireland superimposed for comparison

**Fig. 3:**
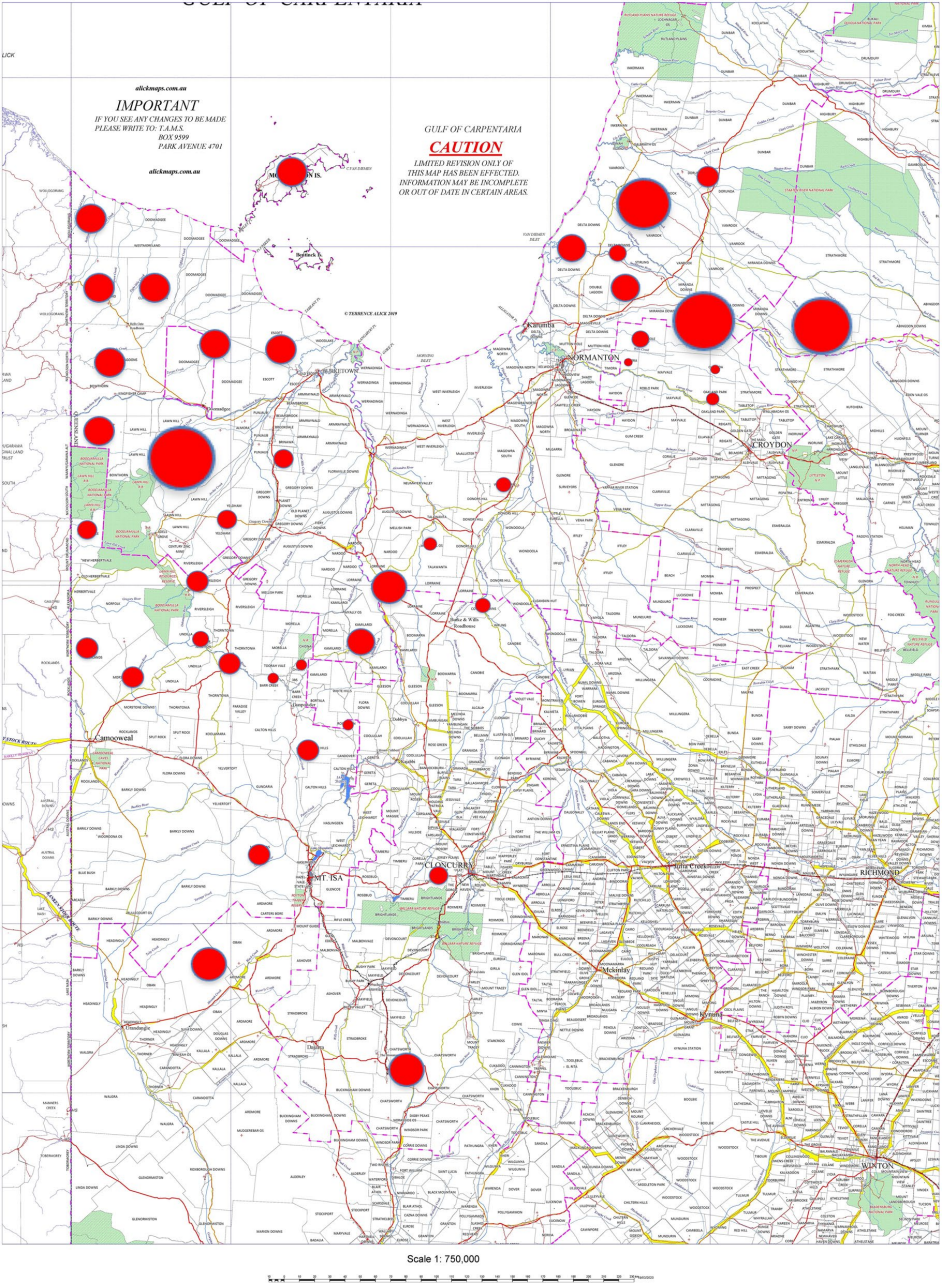
Map of tuberculosis Infected or Restricted Properties Mount Isa Veterinary Division 1987 (from R. Glanville personal records)

In 1987 the author was transferred as Divisional Veterinary Officer to the Mount Isa Veterinary Division in the state of Queensland (Fig. 2), one of 10 veterinary divisions in the state at the time. The Division was roughly double the size of Ireland, with around one million cattle and, at the start of 1987, approximately 51 holdings remained under TB restrictions out of a total of 400 (Fig. 3). Significant eradication work had been undertaken in the years prior to this to reduce the herd prevalence. However, a core of difficult infected properties or areas remained. The region was characterised by very large cattle farms (normally referred to as stations or properties), rugged country, many poor roads and poor communications. For example, many stations had no conventional telephone, relying on radio communications.

The late 1980’s was a crucial time in the program leading up to a target declaration of Impending Freedom from TB by 1 January 1990, which required meeting the criteria for declaration [3]. These criteria, as outlined in the BTEC Standard Definitions and Rules (SDRs) [5] were: no Infected (IN) or Restricted (RD = has achieved one whole herd clean test) herds remaining at the time of declaration; and the capacity to eradicate any breakdown herds (that is, newly detected infection after declaration) within 24 months of detection. To manage this task the Division had three government veterinarians, 17 stock inspectors (animal health technicians), with tuberculin testing largely contracted to private veterinarians. Aircraft operators, particular helicopters, used for paddock inspections and destocking (depopulation) operations discussed later, were also contracted.

In the Mount Isa Division and many other regions across the north of Australia, a number of challenges were faced to achieve eradication. Firstly, a significant number of infected premises remained and although the TB prevalence had been reduced to relatively low levels after many years of effort, much needed to be achieved over a short period of time. This was exacerbated by the relatively short cattle management season dictated by weather. That is, the summer months were too hot to work cattle, plus inaccessibility during the wet season, meant that cattle could only be mustered once or twice per year for operations such as tuberculin testing. Consequently, cattle were often relatively wild and lack of infrastructure, particularly fences, on some properties meant that in some parts of the region the cattle were essentially feral. All of this combined meant that it was difficult to achieve 100 percent musters (rounding up) of cattle for testing and/or depopulation. To overcome the latter issue, two key measures were utilised. Firstly, a mustering subsidy was paid to owners to cover the additional costs of achieving close to 100 percent musters, compared with a normal ‘commercial muster’ (which may have been only 80–90 percent). Secondly, paddocks were inspected by stock inspectors in helicopters to confirm the efficiency of the muster. Any unmustered cattle that were found were generally shot. The region also featured some large areas with feral cattle populations that had to be controlled and/or depopulated.

The mechanism to achieve eradication at a property level was an approved property program (APP) for each of these large cattle stations, tailored to accommodate individual circumstances. The APP was a legal agreement between the property owner and the government agency, and included: property details and it’s TB history; a summary of the eradication program for the coming year and any problems anticipated; the program details, which generally was organised by epidemiological group and/or paddock/area on the property and included animal identification requirements, whether the group was to be tested or depopulated or a combination of both; inspection requirements; movements of cattle within the property; their disease status progression; assistance measures that would apply; and any other general program conditions.

A key issue was to recognise the limitations of the tuberculin test. The test used was the single intradermal caudal fold test, as it was the only one that was practical to use under the circumstances. The gamma interferon test was used late in the program, although it did present practical difficulties and the TB prevalence was so low by this time that it was virtually impossible to judge its value. The sensitivity of the single caudal fold test was at-best 70 percent [6], although often much less in cattle that were often nutritionally challenged towards the end of the cattle season (August–September) or were old. Under these conditions and given the time frames imposed by the program, a traditional ‘test & slaughter’ approach would not have achieved program objectives. Hence, the tuberculin test was used to indicate presence of infection and/or provide confidence of lack of infection. When infection was found in an epidemiological group (normally a paddock of animals where mob integrity was maintained), the group was depopulated, normally by consignment to an abattoir. The depopulation of infected groups, herds or age cohorts was essential to meet the target for Impending Freedom. The philosophy in some ways became ‘only test cattle that you are reasonably confident are free of TB’. Further, testing veterinarians were encouraged to maximise sensitivity (‘read them hard’) and not be concerned about the test specificity. All reactors were destroyed and post-mortem examinations performed, with the owners paid fair compensation. Often, ‘no visible lesion’ (NVL) reactors were also sampled for microbiological examination. Program managers became concerned when the NVL rate for a particular veterinarian became low, indicating a likelihood that small reactions or swellings were being missed.

To Illustrate how the program worked on the ground, three case studies are presented.

## Case study 1 – large herd with long history of infection

This was a large (approximately 90 km by 110 km) cattle station towards the south of the region. It had a carrying capacity of around 30,000 head and was divided into multiple paddocks. Management of such an enterprise is complex, with the annual mustering program having to be carefully orchestrated. This property had a long history of infection, primarily in the western parts but had reached a confirmed free one status (CF1—three whole herd clear tests at least 6 months apart) by 1986, through a program of TB testing and targeted depopulation. However, after a change in ownership, TB was detected in again in 1987 in three animals consigned to the abattoir. An analysis was conducted of the internal epidemiological relationships between groups of cattle on the property, which led to an intensive program of testing and targeted depopulation (either on an age or paddock basis). Overall, 17 TB infected animals were detected between 1987 and 1989, with all infected groups being depopulated. The remaining groups had regained CF1 status through testing by the end of 1989, with approximately 20,000 head remaining. Further whole herd monitoring tests were required in subsequent years, with no further infection detected.

## Case study 2 – the need to maintain flexibility

This was a much smaller property (approximately 27 km by 10 km), but was situated in very rough country in the hills to the north of the city of Mount Isa. The property had never had TB detected at abattoir, but a number of neighbouring properties had a history of infection and this property had never been tested. To organise the required testing, an initial meeting was held with the owner and he was asked, as was normal, to provide or draw a map of the property showing all relevant management features. How a testing program would be managed would then be discussed. A property map usually showed the boundaries of the property, internal paddock fences, stock handling yards etc. In this case, the owner took a piece of paper and started drawing wiggly lines and each of the lines had names. After some consternation, it was realised that he was drawing gullies (valleys, gorges). In this rough country, property boundaries and fences had little meaning. His cattle lived and grazed in gullies and the country was too dry and rugged for them to live anywhere else for most of the year. Once this was understood, a testing program was able to be formulated in conjunction with the neighbours. Mustering occurred on an area basis, with cattle held in holding paddocks while tested. Mustered areas were inspected by helicopter, with any unmustered cattle shot. No TB was found. However, this example is provided to demonstrate the need to be flexible and adapt to individual circumstances.

## Case study 3 – large area approach

**Fig. 4:**
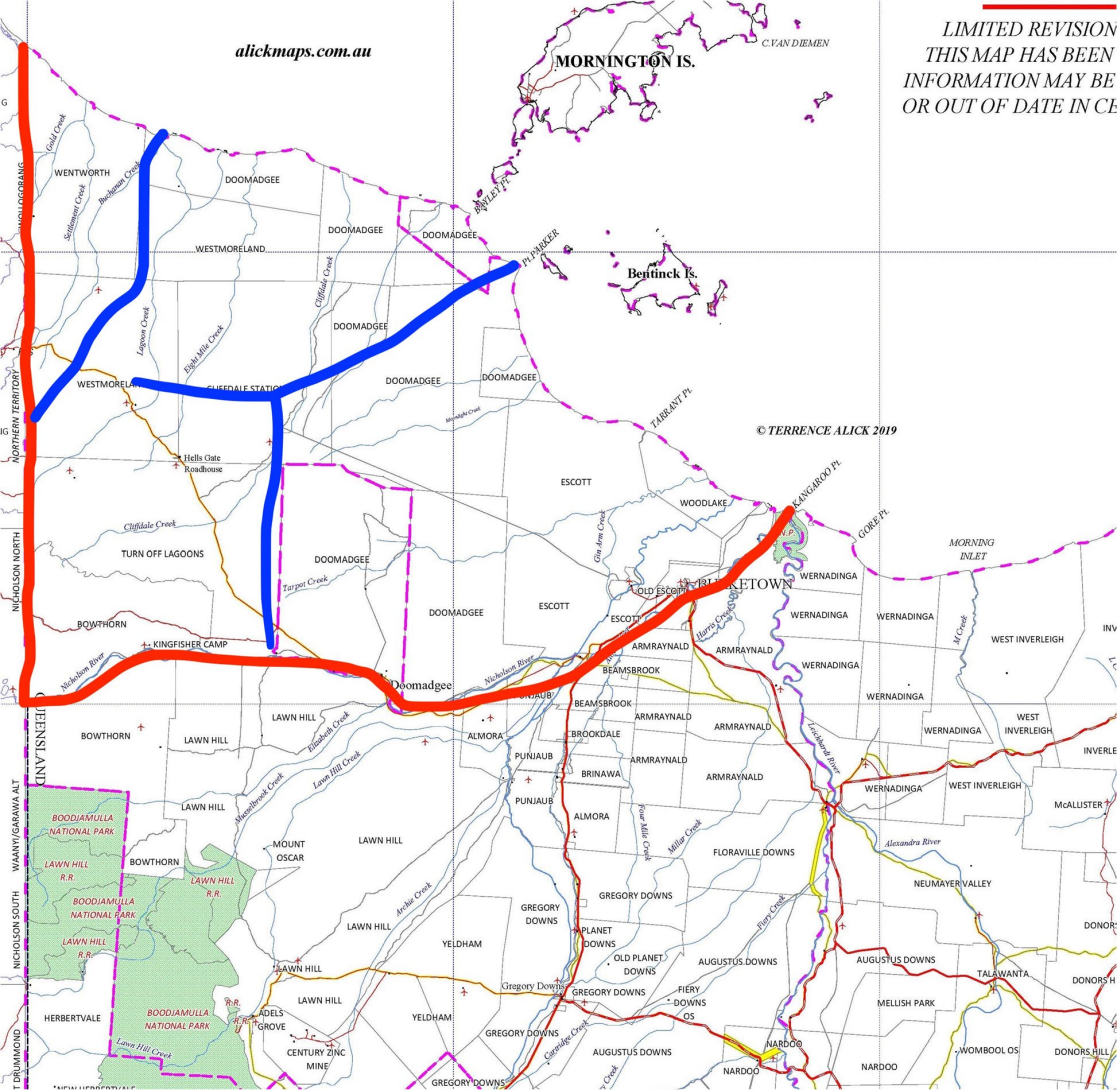
Map of Nicholson Destock Area with strategic fences shown in blue (from R. Glanville personal records)

This example relates to a very large area of about 10,000 square kilometres that in the mid 1980s was largely undeveloped from a cattle production perspective. That is, there were very few stock fences, access roads and cattle handling facilities, despite there being eight land owners. It was referred to as the ‘Nicholson Area’, bound by the Nicholson river to the south, the Northern Territory border to the west and the Gulf of Carpentaria to the north (Fig. 4). Cattle in the area were essentially feral, with commercial operations being limited to harvesting of these animals for consignment to slaughter. The TB infection prevalence was roughly 1 in 1,000. Although this was a very low infection rate, the area was required to be ‘cleaned up’ to meet program targets.

The approach taken was to initially construct a number of ‘strategic’ fences using program funds to break the area into more manageable sections (Fig. 4). These were subsequently utilised in two ways. Firstly, some of the property owners used the strategic fences as a starting point for constructing their own internal paddock fences. They were then able to muster the feral cattle from the areas outside of the paddocks. Younger animals were tuberculin tested and placed into the paddocks, with older animals more likely to have TB or not react to a tuberculin test, being consigned for slaughter. In this way a controlled herd was gradually built up, with introduced bulls used to improved herd quality. Compensation for the destocked animals provided a regular income stream for further property improvements.

The second purpose of the strategic fences was to facilitate area depopulation. There became a point where the mustering of cattle from the ‘outside areas’ became uneconomic. That is, the cost of mustering was greater than the value of animals mustered. At this point, the country was formally ‘handed over’ to the Department, where upon a series of large-scale depopulation operations were conducted over a one to two year period, with all remaining cattle shot from helicopters. This was a large logistical exercise, facilitated by the presence of the strategic fences. Similar operations were conducted in other parts of northern Australia.

## The national program

**Fig. 5:**
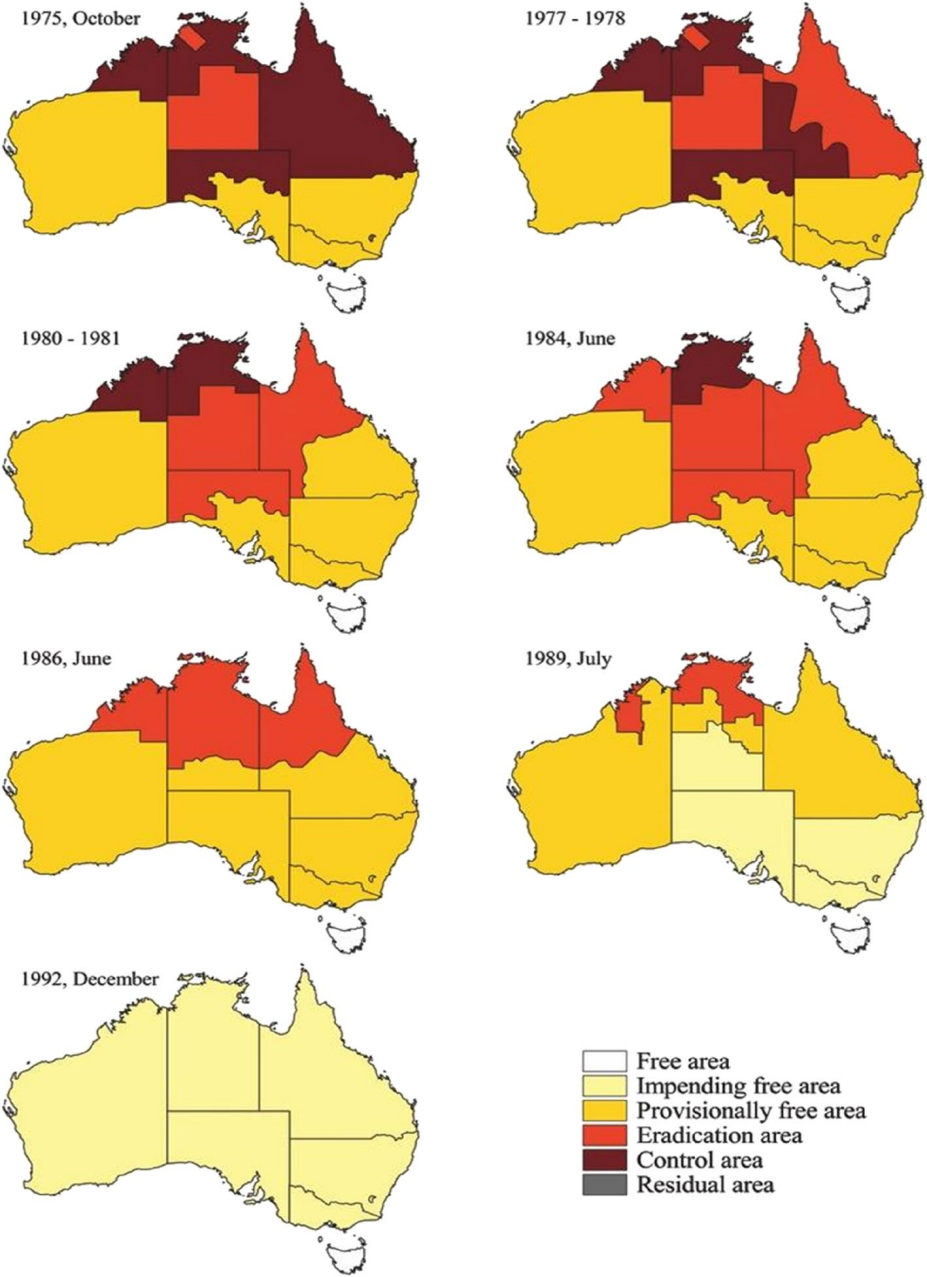
Changes in area classification over time during the Australian tuberculosis eradication campaign. Reproduced from [4]

Through the methods described above, the Mount Isa Division achieved Impending Free status at the end of 1989, as did a number of other similar administrative regions. This was just one of a series of progressive area status changes that drove progress at the national level over time (Fig. 5). In general, TB eradication progressed from the south to the north, rightly or wrongly from the more intensive cattle production areas in the south where TB eradication presented fewer challenges. It should also be noted that much activity had occurred in these areas, greatly reducing TB prevalence, particularly the dairy industry, in the decades prior to BTEC commencing in 1970.

As mentioned earlier, the Standard Definitions and Rules [5] specified various management and technical aspects of BTEC. The elements specified included herd and area status assignment, movement restrictions, testing requirements, animal identification, abattoir monitoring etc. This was crucial to ensure consistency across Australia, given that the responsibility and legislative powers for disease control rest with the state governments, which can act independently. The SDRs were periodically amended over time to reflect changes in methodology or strategic targets [5, 7, 8].

**Fig. 6:**
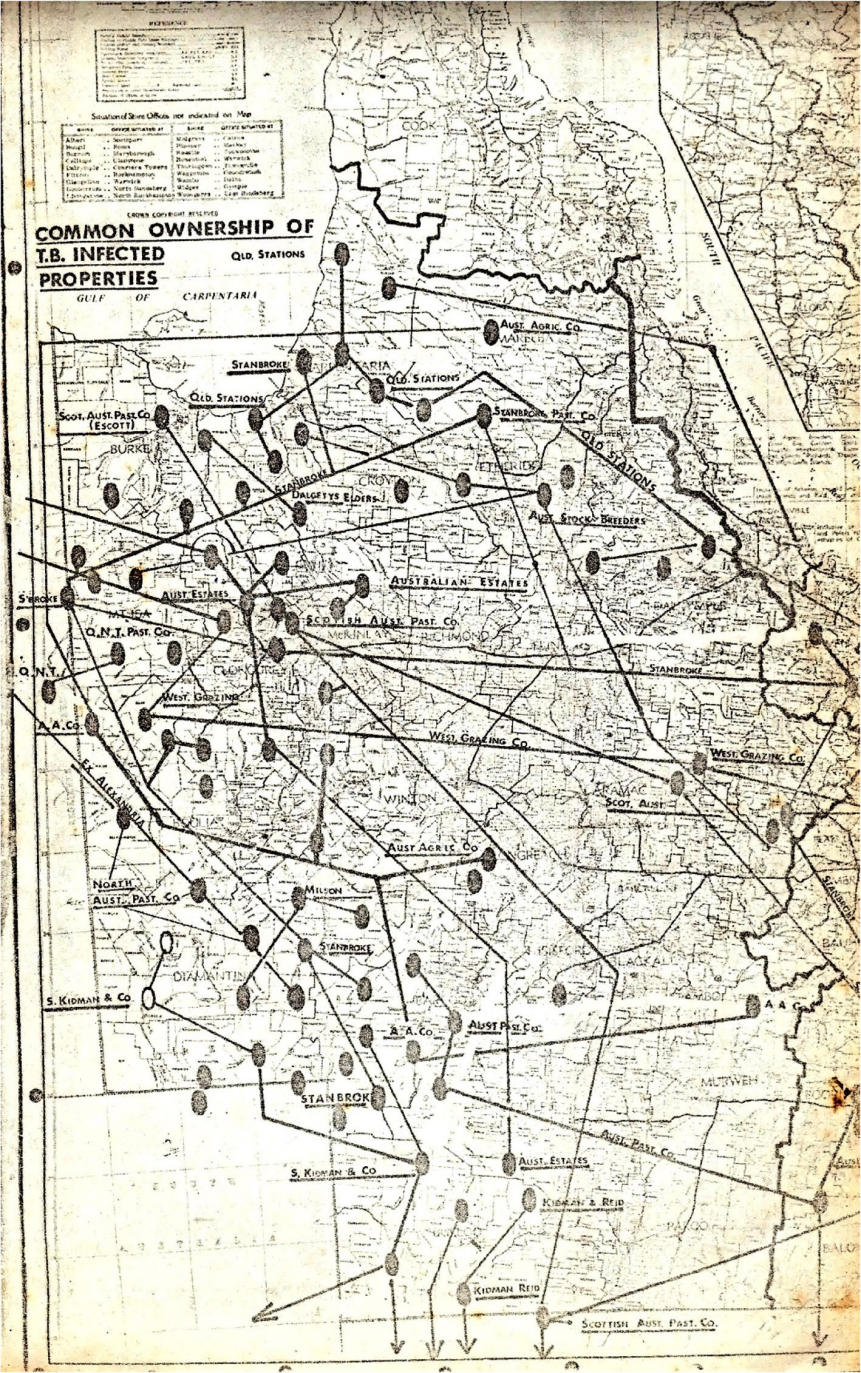
Early map showing common ownership of tuberculosis infected properties from the early 1970s. The inter-linking lines indicate properties under the same ownership, with a general trend of movements between the properties from the north to the south. Supplied by K McCubbin

A key driver for action by cattle owners was the control of cattle movements. Figure 6 shows an analysis conducted in the early 1970s by Dr Owen Brooks of the then Department of Primary Industries of cattle movements within Queensland between TB infected properties under common ownership [9]. It demonstrates the relationships between cattle stations and a general trend of movement of animals from breeding properties in the north to fattening properties in the south. This was a major cause of spread and persistence of TB infection. Hence, risk-based trading was introduced, with the SDRs specifying what movements were allowed as determined by herd and area status. Most significantly, the movement of breeding cattle to other premises was heavily restricted. Allowances were made for movement of cattle for fattening, for example, male cattle to ‘fattening properties’ or ‘approved feedlots’. These latter properties remained under permanent quarantine until depopulated towards the end of BTEC. Movements for slaughter purposes were generally unrestricted. In Queensland, for the duration of BTEC, all cattle movements were required to be accompanied by a movement permit issued by an Inspector who applied the rules that were in place at the time. The movement restrictions were made progressively more stringent as the program progressed. This had the dual effect of stopping the spread of disease and providing a significant incentive for producers to eradicate TB from their herds to regain unrestricted market access.

Another very important element of BTEC was the government – industry partnership that was established formally in 1984 with establishment of the national BTEC Committee [3]. Initially, the program was largely government driven and managed but in the early 1980’s industry leaders became dis-satisfied with their lack of direct involvement. They demanded a greater say in what happened and this led to formation of state and national BTEC committees that established the overall strategic policy and direction. The funding formula also evolved over time to the point where industry paid for 50 percent of the program (initially through slaughter levees and later a transaction levy), the Commonwealth government 20 percent and the State governments 30 percent. Industry then had a strong say in strategic decision making, which galvanised overall industry commitment to the program. One industry leader commonly used the phrase, ‘he who pays, has a say’. It should be noted that the expenditure covered by BTEC funds included direct staff costs, operational costs such as tuberculin testing, vehicles and helicopters, compensation payments and the assistance measures discussed later. Apart from the latter, it did not include consequential losses incurred by cattle producers, such as the loss of production revenue following depopulation or from movement restrictions.

Another mechanism established in some states was Ministerial BTEC advisors. These were people nominated by industry and who were given a brief to be able to visit the various administrative regions, review progress and provide advice on any problem areas. For example, they would sometimes visit a ‘problem property’ with the local Divisional Veterinary Officer to provide valuable advice and assistance to help resolve on-the-ground issues. It is interesting to note that the author observed that the industry representatives were often tougher on their constituents than the government employed staff.

One of the changes to the program influenced by industry involvement was a greater suite of assistance measures to reduce the financial impact of APPs on affected producers. These included market value compensation for animals ordered to be destroyed or depopulated; a mustering subsidy to help cover the additional cost of achieving close to 100 percent musters (as opposed to a commercial muster, which may have been 80–90 percent); a freight subsidy to help cover the cost of freighting replacement stock back to the property; and low interest loans for property improvements such as fences and cattle handing facilities. Taxation concessions were negotiated with the Taxation Department to allow averaging of income over a number of years when large groups were depopulated, and financial counsellors were also made available.

Despite all of the above measures that were used to encourage people to effectively implement property programs, there was the occasional non-complying producer. In these cases, State jurisdictions had the powers within their legislation to take legal action. This occurred on a number of occasions, including Court rulings that enabled the Department to complete the required eradication actions.

Towards the end of BTEC, some innovative methods were implemented to overcome particular problems. This included targeted depopulation of infected, feral water buffalo in local geographical areas based on field surveillance, and the ‘Judas cow’ technique. The latter was developed to deal with areas where it was difficult to find all animals within an area for depopulation, for example, because of very dense vegetation [4]. The technique involved immobilizing a cow from the area of interest with a tranquilizer dart and installing a radio collar. She was then released and periodically tracked. Utilising the natural tendency of cattle to congregate into small social groups, her companions were then shot. This was repeated using one or more such radio collared animals until inspectors were satisfied that the area had been depopulated.

## Following BTEC

BTEC underpinned Australia’s animal health system for many years. Post-BTEC there have been a number of ongoing legacies of the program. The property improvements in the northern industry that were required to achieve eradication have largely persisted, meaning that the beef cattle industry became much more modern and productive. The infrastructure improvements allowed better management of cattle, including use of improved genetics, controlled mating and culling of poor performers, all leading to improved productivity. The partnership approach between government and industry has persisted and is now an imbedded feature of Australia’s biosecurity system. For example, Animal Health Australia (AHA) is a formal industry – government partnership. It is a not-for-profit public company with 34 government and industry members that coordinates a significant number of national animal health programs [10]. One mechanism coordinated by AHA is the Emergency Animal Disease Response Agreement (EADRA) [11] that, again, is a legally binding agreement between the State and Federal governments, and industry organisations for managing emergency disease events. It should be noted that bovine tuberculosis is now listed as an exotic disease under EADRA. During BTEC, an effective animal tracing system was developed using registration of cattle enterprises, tail tags bearing the property’s registration number, fire brands (in some states) and movement documentation. The key elements of this system have been retained and now enhanced through application of animal identification using radio frequency identification devices, together with recording/capture of movements within a national database [12]. This has provided an almost real time cattle tracing capability.

**Fig. 7:**
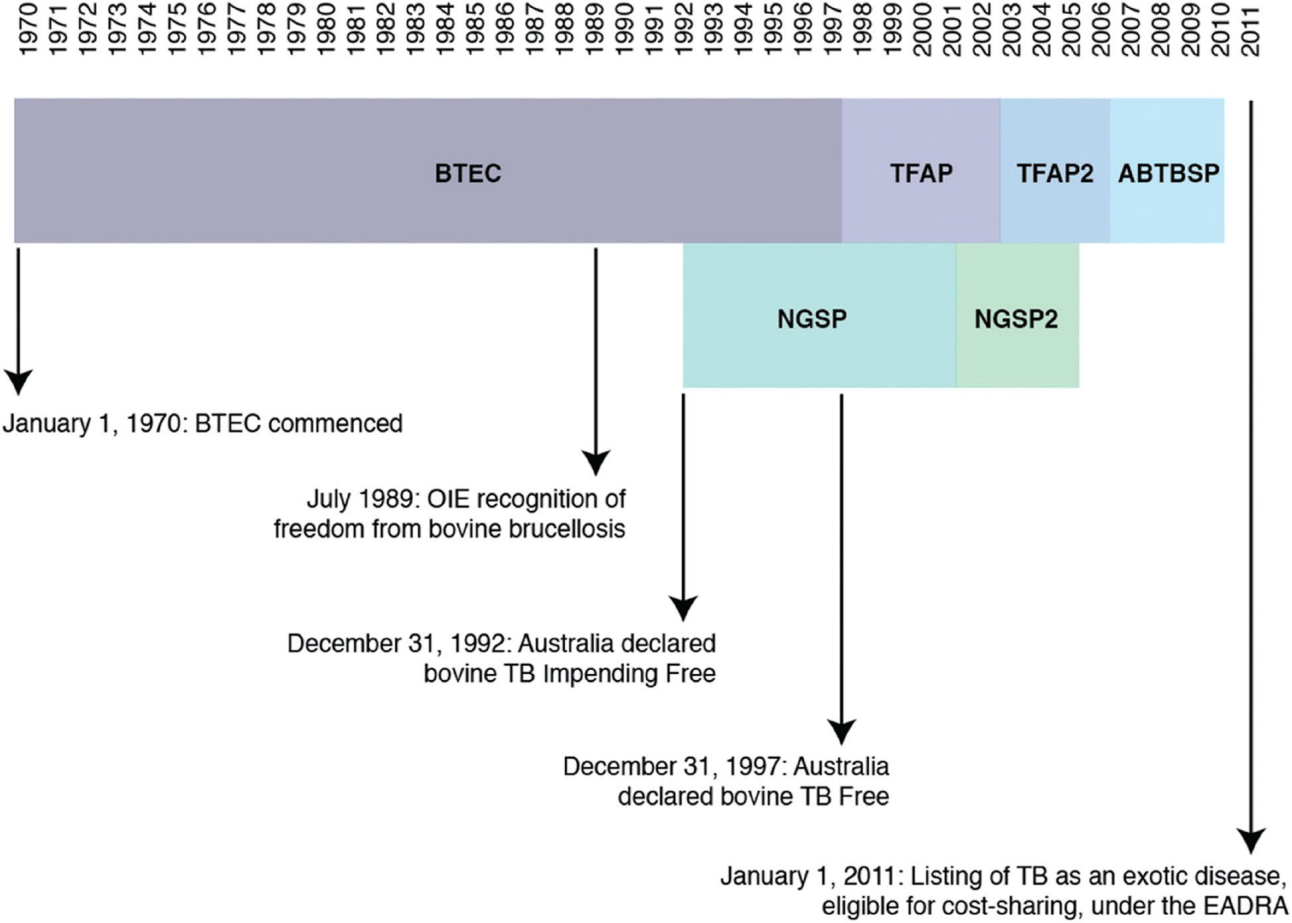
Chronology of key events in the brucellosis and tuberculosis eradication campaign (BTEC) and related programmes. ABTBSP Australian Bovine Tuberculosis Surveillance Project, EADRA Emergency Animal Disease Response Agreement, NGSP National Granuloma Submission Program, OIE World Organisation for Animal Health, TB Tuberculosis, TFAP Tuberculosis Freedom Assurance Programme. Reproduced from [4]

**Fig. 8:**
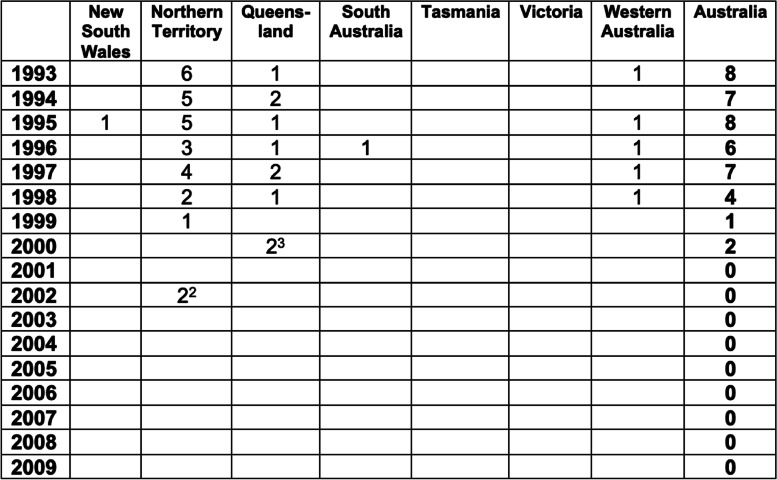
Number of tuberculosis primary case^1^ herds in each Australian State from 1993 to 2009. Compiled using data from [13] and data supplied by John Roberts from the national TB case register ^1^ Primary cases were defined as those detected in case herds. This means a herd in which a case of tuberculosis wasfound, that was previously tested Negative, Monitored Negative or Confirmed Free A secondary case herd was defined as a herd found to be infected with tuberculosis following tracing from a Case Herd. During the same period (1993-2009) there were 11 secondary case herds ^2^ These were two primary cases in adjoining, managed buffalo herds in 2002 in the Northern Territory. Both were found to be residual infection in old cows, with no further infection detected ^3^ One of these two cases did not fully meet the definition of a primary case, as it was detected in secondary case herds after the original herd had been fully dispersed and no longer existed. However, it is listed here for completeness. The last TB detection in cattle in 2002 was in a secondary case herd associated with this case

Following declaration of tuberculosis freedom on 31 December 1997, BTEC concluded and was replaced by the Tuberculosis Freedom Assurance Program and a granuloma submission program, with a major focus on enhancing detection at abattoirs of any remaining residual TB infection [1, 4]. Figure 7 shows a timeline for these programs. Figure 8 indicates that small numbers of infected premises were detected after the date when the whole of Australia reached Impending Freedom status (31 December 1992), as was expected. However, these tended to be very small numbers of animals with encapsulated TB lesions [1]. The last TB lesions in cattle were detected in a secondary case herd in 2002 (see Fig. 8 for a definition of primary and secondary case herds). However, program managers continued to treat these cases seriously and applied stringent measures to ensure that no TB remained.

## The last primary case

This stringent approach is illustrated by the handling of the last officially recognised primary case of TB in cattle which was detected in 2000 [4, 14]. This was a large (579,000 hectare) property carrying a brahman cross herd of approximately 40,000 head. The property had a long history of infection, with greater than 20 percent TB prevalence detected in some consignments of bulls turned off for slaughter during the 1970s. Control and eradication commenced in 1972 using a mixture of tuberculin testing and depopulation. The last case of TB on this property was recorded in 1991, with confirmed free status being achieved by 2000. However, in August and October 2000, two infected cows were detected at abattoir. These were determined to be seven and eight years of age (firebrands for this property included a year of birth brand component) and also property records provided the two paddocks from which they were mustered. In 2001 these two paddocks were compulsorily depopulated, with no TB lesions detected in over 8,000 animals slaughtered. In 2002 all remaining cattle on the entire property that were over five years of age were consigned to slaughter, with the remainder tuberculin tested. All unmustered cattle were shot and autopsies conducted where possible. In all, 9,533 animals were destroyed or consigned for slaughter that year, with 33,169 head tuberculin tested. No TB lesions were detected. This entire exercise was repeated in 2003/4 and again in 2006, with no TB infection detected. Hence, through this comprehensive program of depopulation and testing, no further TB was detected. This provided confidence that no further TB infection remained.

A similar process was followed on all other breakdown properties following declaration of Impending Freedom, often involving depopulation of the entire herd. An incentive program was subsequently introduced to encourage accelerated turn-off to abattoir of any remaining cattle on these properties that had not been depopulated, thus providing further confidence that the disease had been eradicated.

## Conclusions

This paper highlights a number of key design features of the overall Australian program that led to its success. Foremost, there was commitment from both government and industry, with clear and agreed outcomes and targets. This was supported by robust program planning and management, which included timelines and measures of progress. These aspects were supported by appropriate funding through a cost shared model. There were agreed, national technical standards that all jurisdictions implemented for national consistency (including laboratory support) and these worked hand in hand with strict controls, particularly around animal testing, movement controls based on herd and area status and targeted depopulation of infected cohorts. These controls had a solid legal backing. A very strong surveillance system to identify infected herds was in place, with a particular emphasis on abattoir monitoring. This required an effective cattle tracing system. Finally, a suite of assistance measures was in place to minimise the financial impact on individual producers.

Across all of these success factors there was an over-arching culture of persistence and being prepared to be tough where required. Another intangible success factor was possibly the Australian cultural tendency to respect our institutions and laws. This was highlighted in a recent Australian survey, despite an image of Australia that is perhaps the opposite [15].

## Data Availability

Data sharing is not applicable to this article as no datasets were analysed. However, the statistics quoted are either from original records or published papers and can be quoted.

## Abbreviations

AHA: Animal Health Australia
APP: Approved property program
AUD: Australian dollar
BTEC: Brucellosis and Tuberculosis Eradication Campaign
CF1: Confirmed Free One (herd status)
EADRA: Emergency Animal Disease Response Agreement
IN: Infected (herd status)
Km: Kilometre
NVL: No visible lesion
RD: Restricted (herd status)
SDRs: Standard Definitions and Rules
TB: Tuberculosis
USA: United States of America
